# Correction: High atherogenic risk concomitant with elevated HbA1c among persons with type 2 diabetes mellitus in North Ethiopia

**DOI:** 10.1371/journal.pone.0300773

**Published:** 2024-03-13

**Authors:** Hagos Amare Gebreyesus, Girmatsion Fisseha Abreha, Sintayehu Degu Besherae, Merhawit Atsbha Abera, Abraha Hailu Weldegerima, Aregawi Haileslassie Gidey, Afework Mulugeta Bezabih, Tefera Belachew Lemma, Tsinuel Girma Nigatu

After publication of this article [1], the corresponding author provided the underlying data supporting the published results. With the provision of these data, available in S1 Data with this notice, this article [1] complies with PLOS’ Data Availability Policy.

## Supporting information

S1 Data Underlying dataset supporting the results in this article [1]. (SAV)
