# Supplementary material for: High atherogenic risk concomitant with elevated HbA1c among persons with type 2 diabetes mellitus in North Ethiopia
Source: PLoS One. 2022 Feb 1;17(2):e0262610. doi: 10.1371/journal.pone.0262610 (PMC8806058; doi:10.1371/journal.pone.0262610)
Supplement: S1 Appendix — (DOCX) [file pone.0262610.s001.docx]

**Structured questionnaire for assessing demographic background, diabetic follow up and nutrition information and anthropometric and biochemical data of adults with type 2 diabetes mellitus**

1. **Demographic background of participants**

| **No** | **Question** | **Response** | | **Go to/Skip** |
| --- | --- | --- | --- | --- |
| 101 | Age |  | _____ |  |
| 102 | Sex | Male  Female |  |  |
| 103 | Residence | Urban  Rural |  |  |
| 103 | Nationality |  | ________ |  |
| 104 | Ethnicity | Tigrian  Amhara  Oromo  Afar  Other (specify) |  |  |
| 105 | Religion | Orthodox  Muslim  Catholic  Protestant  Others (specify) |  |  |
| 106 | Marital status | Single  Married  Widowed  Divorced |  |  |
| 107 | Educational status | Illiterate  Able to read and write  Primary school  Secondary school  College graduate or above |  |  |

1. **Diabetic follow up and nutrition information of persons with type 2 diabetes mellitus**

| 101 | How long has it been since you diagnosed with T2DM? | ------- years |  |  |
| --- | --- | --- | --- | --- |
| 102 | How long has it been since you begun DM follow-up? | --------years |  |  |
| 103 | How many times do you visit the diabetic follow up clinic in the last six months? | -----------/year |  |  |
| 104 | How do you control your blood glucose? | By diet  By exercise  By medication  By diet and exercise  By diet and medication  By exercise and medication  By diet, exercise & medication |  |  |
| 105 | Type of medication for diabetes | Metformin  Glibenclamide  Metformin & Glibenclamide  Insulin  Insulin + Metformin | **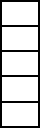** |  |
| 106 | Treatment adherence | Poor  Moderate  High | **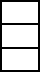** |  |
| 107 | Do you have other co morbidities or complications | Yes  No |  | If No skip 108 |
| 108 | What type? specify | Hypertension  Retinopathy  Neuropathy  Nephropathy  Cardiovascular diseases  Others (specify) |  |  |
| 109 | Did you get any nutritional education or advice regarding dietary control of diabetes so far? | Yes  No |  | If the response is No,skip Q110-111 |
| 110 | Who gave it to you and where?  (More than one response is allowed) | Doctor  Nurse  Nutritionist  Other team of health professionals as a community service |  |  |
| 111 | How was it given? More than one response is possible | Individually  In Group  Orally  In written |  |  |

1. **Anthropometric and biochemical data of persons with type 2 diabetes**

| Measurement | Value | Date | Measurement | Value 1 | Value 2 | Average | Date |
| --- | --- | --- | --- | --- | --- | --- | --- |
| HbA1c |  |  | Waist circumference (cm) |  |  |  |  |
| Total cholesterol |  |  | Height(m) |  |  |  |  |
| Triglyceride |  |  | Weight(kg) |  |  |  |  |
| LDL |  |  |  |  |  |  |  |
| HDL |  |  |  |  |  |  |  |

BMI……………… Waist-to-hip ratio…………

Blood pressure in __________ mmHg
