# Supplementary material for: High atherogenic risk concomitant with elevated HbA1c among persons with type 2 diabetes mellitus in North Ethiopia
Source: PLoS One. 2022 Feb 1;17(2):e0262610. doi: 10.1371/journal.pone.0262610 (PMC8806058; doi:10.1371/journal.pone.0262610)
Supplement: S2 Appendix — (DOCX) [file pone.0262610.s002.docx]

**ዓይነት 2ተ ናይ ሽኮር ሕማም አብ ዘለዎም ሰባት ዝግበር ናይ ግላዊን ማሕበራዊን ኩነታት፣ ኽትትል ሕማም ሽኮርን ሓበሬታ ኣብ ስነ−መዐዛን፣ ናይ ዉሽጢ ሰውነት ኬሚካላትን ደጋዊ አካላትን መረዳእታ ዳህሰሳ ዘገልግል ዝተመዓራረየ መጠይቕ**

**1. ግላዊን ማሕበራዊን ኩነታት**

| **ተ.ቑ** | **ሕቶ** | **መማረፅታት መልሲ** | | **ቐፅሉ/ይዝለሉ** |
| --- | --- | --- | --- | --- |
| 101 | ዕድመ | ------------- |  |  |
| 102 | ፆታ | ተባዕታይ  አንስታይ |  |  |
| 103 | ዝነብሩሉ ቦታ | ከተማ  ገጠር |  |  |
| 104 | ዜግነት | ----------------- |  |  |
| 105 | ዓሌት | ትግራዋይ  አምሓራይ  ኦሮሞ  ዓፋር  ካልእ (ይግለፁ) |  |  |
| 106 | ሃይማኖት | ኦረቶዶክስ  ሙሰሊም  ካቶሊክ  ፕሮቲስታንት  ካልእ (ይግለፁ) |  |  |
| 107 | ኩነታት ሓዳር | ዘየእተወ/ት  ዘእተወ/ት  ዝፈትሐ/ት  ብሞት ዝተፈለየቶ/ያ |  |  |
| 108 | ኛይ ትምህርቲ ደረጃ | ምንባብን ምፅሓፍን ዘይኽእሉ/ላ  ምንባብን ምፅሓፍን ዝኽእሉ/ላ  ቀዳማይ ደረጃ ትምህርቲ ዘጠናቐቐ/ት  ካልአይ ደረጃ ትምህርቲ ዘጠናቐቐ/ት  ኮሌጅን ካብኡ ንላዕልን ዘጠናቐቐ/ት |  |  |

**2.ኽትትል ሕማም ሽኮርን አፍልጦ ጥዕናዊ አመጋግባን**

| 301 | 2ይ ዓይነት ናይ ሽኮር ሕማም ከምዘለዎም ብሕክምና ዝፈለጡሉ አብ ክንደይ ዓመቶምነይሩ? | ------------ |  |  |
| --- | --- | --- | --- | --- |
| 202 | ናይ ሽኮር ሕማም ኽትትል ካብ ዝጅምሩ ክንደይ ዓመት ገይሮም? | -------------- |  |  |
| 303 | አብ ዝሐለፉ 6ተ አዋርሕ ክንደይ ግዜ ክትትል ገይሮም? | --------------- |  |  |
| 304 | አብ ደሞም ዘሎ ናይ ሽኮር መጠን ንምቑፅፃር ዝጥቀምሉ መንገዲ | ብምግቢ  ብአካል ምንቅስቓስ  ብመድሓኒት  ብምግቢን አካል ምንቅስቓስን  ብምግቢን ብመድሓኒት  ብአካል ምንቅስቓስን ብመድሓኒትን  ብምግቢአካል ምንቅስቓስን ብመድሓኒትን |  |  |
| 305 | ሐዚ ዝወስድዎ ዘለዉ ናይ ሽኮር ሕማም መቆፃፀሪ መድሓኒት | ሓደ ዝወሓጥ መድሓኒት ጥራሕ  2ተን ካብኡ ንላዕሊን ዝወሓጡ መድሓኒታት  ኢንሱሊን  ዝወሓጥ መድሓኒትን ኢንሱሊንን ብጣምራ |  |  |
|  | ኽትትል መድሓኒት | ልዑል  ማእኸላይ  ትሑት | \|  \| \| --- \| \|  \| \|  \| |  |
| 306 | ካልኦት ምስ ሕማም ሽኮር ተታሓሓዝቲ ሕማማት አለዉዎም ’ዶ? | እወ አለኒ/ዉኒ  አይ የብለይን |  |  |
| 307 | ካብዞም አብ ጎኒ ዝተዘርዘሩ አየነኦም እዮም? | ደም በዝሒ  ናይ ዓይኒ ሕማም  ናይ መትኒ ሕማም  ናይ ኩላሊት ሕማም  ናይ ልብን ሰራውር ደምን ፀገም  ካልእ (ይጥቀሱ) |  |  |
| 308 | ሕማም ሽኮር ንምቁፅፃር ዝሕግዝ ናይ ስነ-ምግቢ ኣስተምህሮ ተዋሂብዎምዶ ይፈልጥዶ? | እወ ተዋሂቡኒ  ተዋሂቡኒ አይፈልጥን |  | ኣይተውበኒን ናብ ቁ. 3 |
| 309 | ብመን ተዋሂብዎም? | ብዶክተር  ብነርስ  ብናይ ስነ-ምግቢ ብዓል ሞያ  ብካልእ ናይ ሕክምና ቡድን ከም ማሕበራዊ ግልጋሎት አስተምህሮ |  |  |
| 310 | ብኸመይ መንገዲ ተዋሂቡ? | ንግለይ ብቃል ተዋሂቡኒ  ንግለይ ብፅሑፍ ተዋሂቡኒ  ብጉጅለ ብቃል ተዋሂቡና  ብጉጅለ ብፅሑፍ ተዋሂቡና |  |  |

1. **ናይ ሰውነት ዉሽጢ ኬሚካላትን ደጋዊ አካላትን ዳታ**

| ዓቐን | ልክዒት | ዕለት | ዓቐን | ልክዒት 1 | ልክዒት 2 | ማእኸላይ ውፅኢት | ዕለት |
| --- | --- | --- | --- | --- | --- | --- | --- |
| ሂሞግለቢ ኤ 1 ሲ/ፋስቲንግ ግልኮስ |  |  | ናይ ከብዲ ፅፍሓት (ሰ.ሜ) |  |  |  |  |
| ቶታል |  |  | ናይ መቐመጫ ፅፍሓት(ሰ.ሜ) |  |  |  |  |
| ትራይግላይሰራይድ |  |  | ቑመት (ሜ) |  |  |  |  |
| ልዲል |  |  | ክብደት(ኪ.ግ) |  |  |  |  |
| ችዲል |  |  |  |  |  |  |  |

ቢ.ኤም.አይ……………… ፅፍሓት ከብዲ-አብ ልዕሊ መቐመጫ…………

ፀቕጢ ደም__________
